# A Rare Case of Extra-renal Clear Cell Renal Cell Carcinoma

**DOI:** 10.7759/cureus.60246

**Published:** 2024-05-13

**Authors:** Napoleon Moulavasilis, Gerasimos Tsourouflis, Periklis Anastasiou, Konstantinos Douroumis, Alexios Terra, Ifaistion M Palios, Ioannis Anastasiou

**Affiliations:** 1 First Department of Urology, National and Kapodistrian University of Athens, Athens, GRC; 2 Second Department of Propaedeutic Surgery, National and Kapodistrian University of Athens, Athens, GRC; 3 Department of Urology, University of Ioannina, Ioannina, GRC

**Keywords:** mesonephric embryonal remnants, colonic fossa, clear cell carcinoma, extra-renal rcc, renal cell carcinoma

## Abstract

Renal cell carcinoma (RCC) is the predominant solid lesion found in the kidney. Extra-renal RCC is a rare entity. We present the case of a 75-year-old male with an incidentally discovered mass in the right iliac fossa. The patient underwent active surveillance because a percutaneous biopsy revealed a mesenchymal neoplastic lesion of benign biological behavior. As the mass had high growth rates, a decision for open surgical exploration and excision was made. The pathology results indicated clear cell renal carcinoma, and negative results on ^18^F-FDG whole-body positron emission tomography-computed tomography (PET/CT) established the diagnosis of extra-renal clear cell RCC. Similar types of neoplasms are extremely rare and are estimated to have developed primarily in mesodermal embryonic remnants. Clinicians should be aware of this rare entity as its diagnosis is challenging and is based on pathology.

## Introduction

Renal cell carcinoma (RCC) constitutes about 3% of all cancer cases [1]. It stands as the predominant solid lesion found in the kidney, making up roughly 90% of all kidney malignancies [2]. Established risk factors, such as smoking, obesity, and hypertension, have been confirmed. Nevertheless, there is currently no evidence endorsing primary screening for the general population [3].

The extra-renal localization of RCC refers to the presence of RCC in a site outside the kidneys, and this is an uncommon occurrence documented only twice in the existing literature [4,5]. This condition is deemed a highly infrequent pathology, likely originating from mesonephric remnants. We present a case of extra-renal RCC incidentally found at the right iliac fossa. As this condition is very rare, it is important for clinicians to be aware of this diagnosis.

## Case presentation

**Figure 1:**
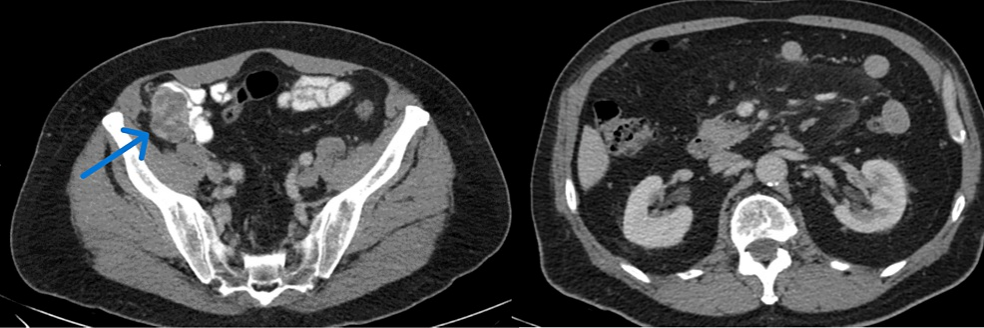
Left: Computed tomography imaging of the mass (axial plane). Right: Computed tomography imaging of the kidneys, without evidence of mass (axial plane). The arrow shows the enhanced mass located in the right iliac fossa, with a diameter of 4.3 cm.

A 75-year-old male was referred to our department due to a tumor incidentally discovered at the right iliac fossa by ultrasound (US). The ultrasound was performed for post void residual volume measurement, for benign prostatic hyperplasia evaluation. The patient did not complain about any symptoms and the clinical examination was unremarkable. The patient did not have a history of smoking, hypertension, metabolic syndrome, or obesity nor had first-degree relatives with a history of RCC. A subsequent computed tomography (CT) scan of the abdomen revealed a mass with a diameter of 4.3 cm in the right iliac fossa. The mass was located anterior to the colonic vessels, with characteristics suggestive of lymphadenopathy (Figure 1). The examination was unremarkable for other abnormal findings (Figure 1).

The unusual position, along with the imaging characteristics of the tumor, prompted the decision for histological diagnosis through percutaneous biopsy. The histological examination revealed a mesenchymal neoplastic lesion of benign biological behavior, with vascular origin.

**Figure 2:**
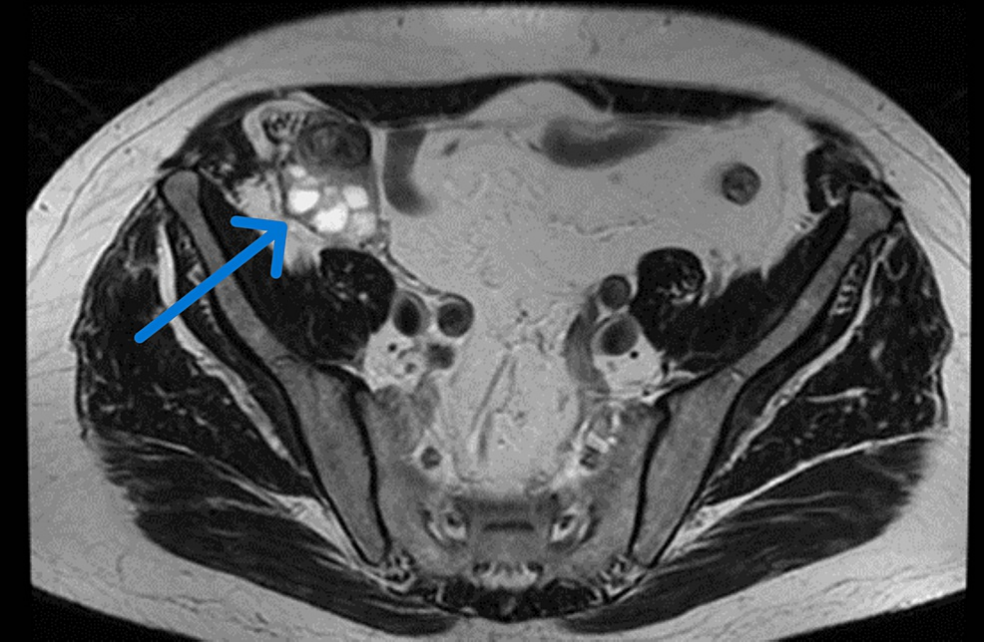
Follow-up MRI (T1-weighted) imaging of the mass. The arrow shows the mass in the right iliac fossa, measuring 5.3 cm in diameter.

As the pathology findings were suggestive of a benign tumor the patient underwent active surveillance. As part of the follow-up, the patient underwent magnetic resonance imaging (MRI) of the upper and lower abdomen after three months. The findings reported a lobulated formation with cystic and solid components, demonstrating intense and heterogeneously enhanced intravenous contrast uptake, measuring 5.3 cm in diameter. It was noted that the mass was in contact with the right colonic flexure, the ascending colon, and the ileocecal coils, without evidence of invasion. Also, the mass was in contact with the right spermatic artery and the right spermatic cord (Figure 2). Both kidneys had normal size, location, and enhancement.

**Figure 3:**
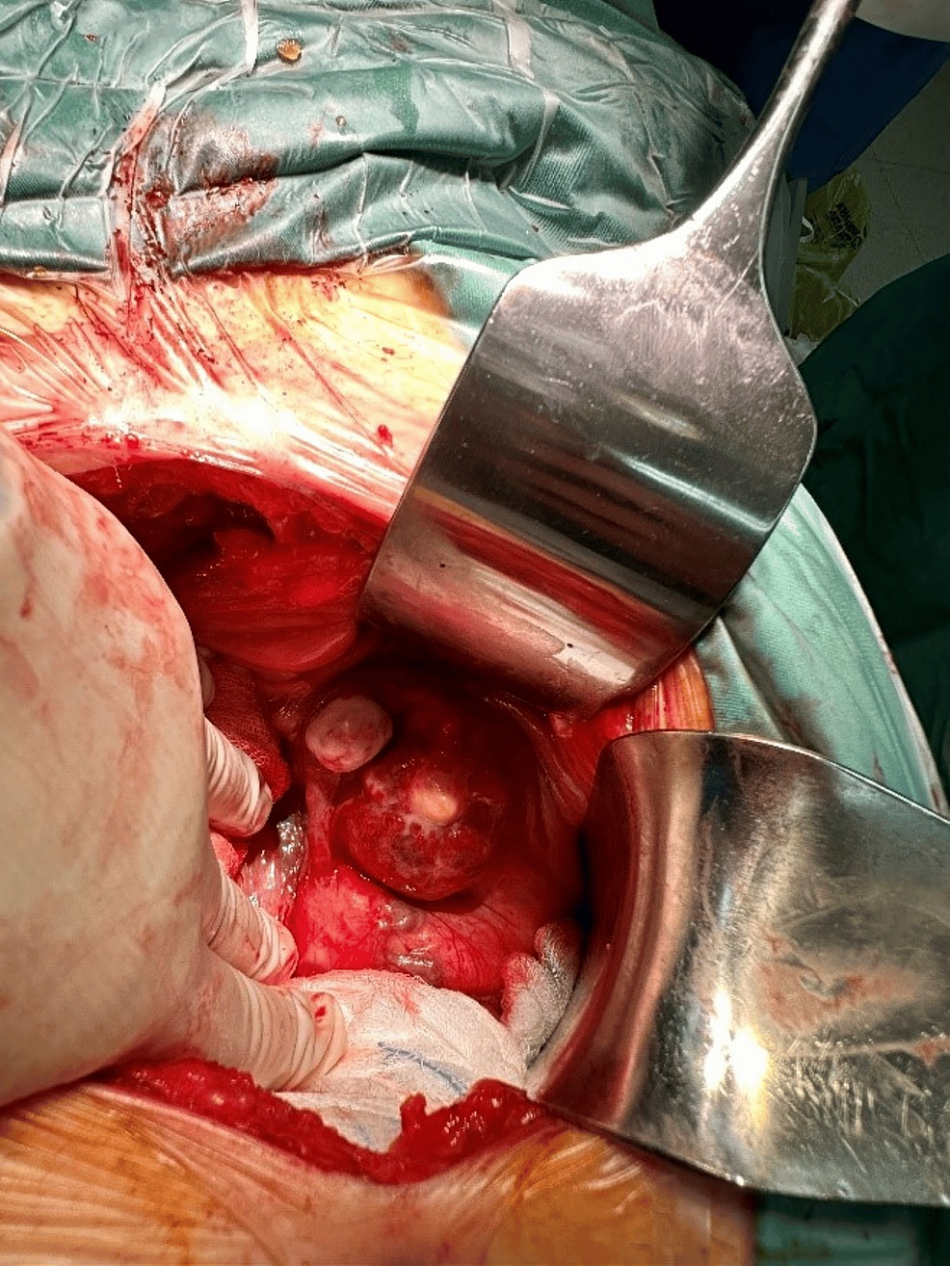
Intraoperative view of the mass.

**Figure 4:**
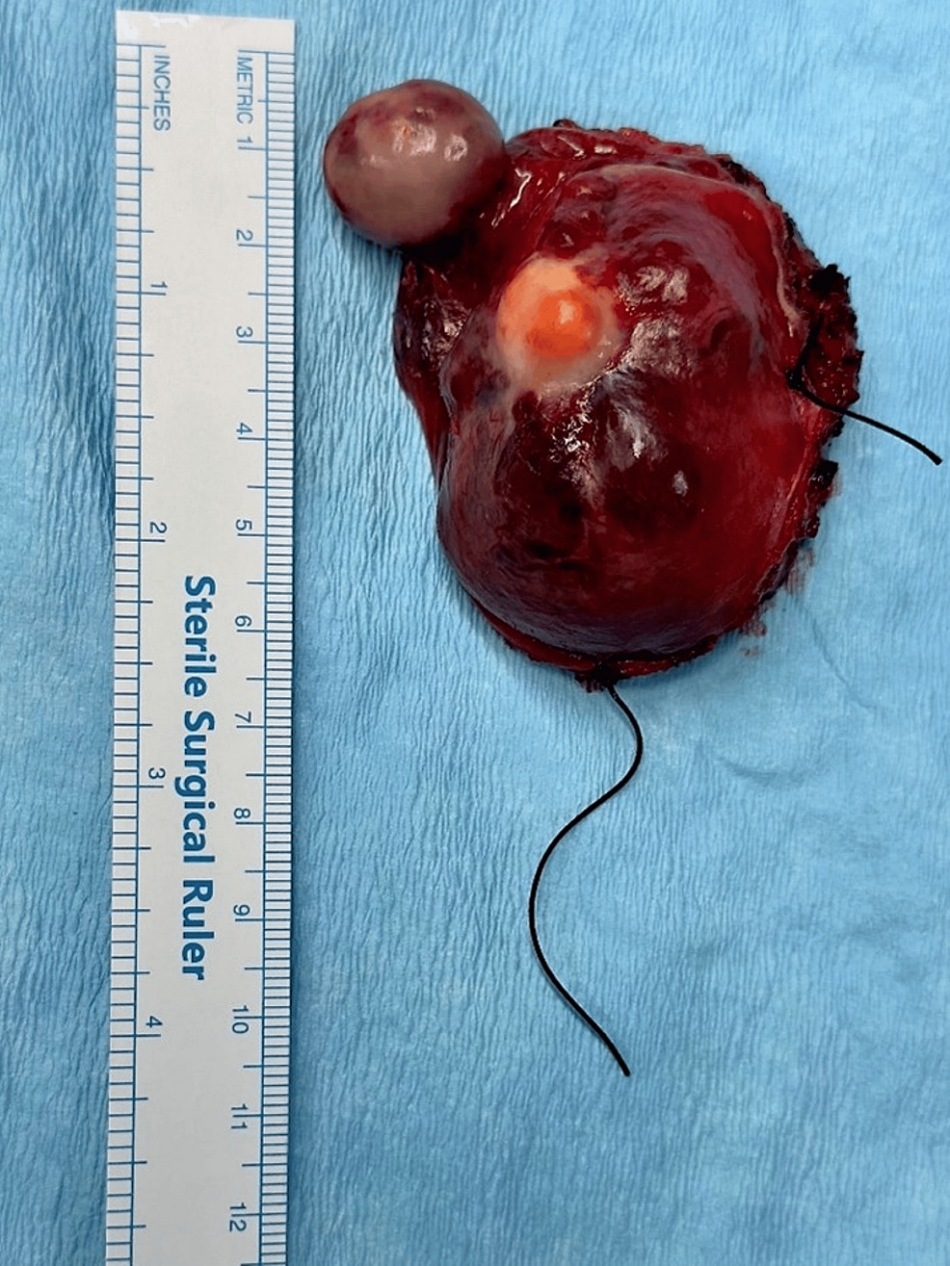
The mass after the excision.

After presenting the case to our hospital's oncology multidisciplinary team (MDT), a decision was made to proceed with open surgical excision of the aforementioned mass. As the mass was close to the colonic vessels, we opted for open surgical exploration rather than laparoscopic, to have better control in case of hemorrhage. Under general anesthesia, surgical exploration and excision were performed, achieving macroscopically negative margins of the mass. Intraoperatively, the mass had features consistent with a solid tumor that did not infiltrate surrounding tissues (Figures 3, 4). The post-operative course was uneventful, and the patient was discharged after three days.

The tissue submitted for pathologic reevaluation included a histological section of a malignant tumor with morphological features of a transmucosal neoplasm with an immunophenotype supporting the diagnosis of RCC (Vimentin {+}, CD10 {+}, CK7 {+}, focal in a small percentage of cells, CK8/18 {+}, PAX-8 {+, in a few cells}, CA-IX {+}, MART-1 {-}, SOX-10 {-}, HMB-45 {-}, CD34 {-}, Bcl2 {-}). The cell proliferation marker Ki67 was found positive in <1% of neoplastic cells (nuclear distribution). These features were indicative of clear cell renal carcinoma.

A subsequent 18F-FDG whole-body positron emission tomography-computed tomography (PET/CT), two months after surgery, was negative for residual tumor or renal involvement. As such, a diagnosis of extra-renal clear cell RCC was established. An MDT meeting was performed after these results, where the option of adjuvant treatment was discussed. As there is no evidence that supports the adjuvant treatment in this scenario, along with the negative PET scan, the MDT supported the follow-up of the patient, without adjuvant treatment. The patient is currently under follow-up, and a CT scan of the abdomen is going to be performed at the six-month follow-up.

## Discussion

The diagnosis of most renal tumors often occurs incidentally during abdominal US or CT scans conducted for unrelated medical reasons [6]. Renal masses are categorized as either solid or cystic based on imaging observations.

In the case of solid renal masses, a crucial criterion for distinguishing malignant lesions is the presence of enhancement [7]. Imaging protocols typically include unenhanced scans, early arterial phase scans, and parenchymal phase scans with intravenous contrast material to highlight any enhancement. In CT imaging, the enhancement in renal masses is assessed by comparing Hounsfield units (HU) before and after the administration of contrast [7]. In this case, the mass was enhanced during the parenchymal phase, but due to its uncommon location, RCC was not included in the differential diagnosis.

The embryology of kidneys involves their development during fetal development. The kidneys originate from the intermediate mesoderm, specifically the nephrogenic cord. This developmental process initiates with the formation of the pronephros, a transient structure that undergoes regression later on [8].

After the regression of the pronephros, the mesonephros undergoes development, comprising mesonephric tubules and associated glomeruli. However, similar to the pronephros, the mesonephros also experience regression over time [8].

The permanent kidneys, referred to as metanephros, commence development around the fifth week of gestation. The metanephros originate from two primary components: the ureteric bud and the metanephric mesenchyme. The ureteric bud extends from the mesonephric duct and gives rise to the kidney's collecting system, including the renal pelvis, calyces, and collecting ducts [8].

Conversely, the metanephric mesenchyme contributes to the formation of nephrons, the functional units of the kidney responsible for filtration and urine production. The metanephric mesenchyme undergoes an intricate process of differentiation and interaction with the ureteric bud to give rise to nephrons, comprising glomeruli, proximal tubules, loop of Henle, and distal tubules [8].

As development advances, the ureteric bud undergoes branching and elongation, ultimately giving rise to the collecting duct system, while the metanephric mesenchyme undergoes differentiation into nephrons. This intricate process culminates in the formation of two fully functional kidneys, crucial for maintaining fluid and electrolyte balance, filtering waste products, and regulating blood pressure [8].

Disruptions or abnormalities occurring during kidney development can result in congenital kidney disorders or malformations. Some mesonephric structures may persist into fetal and postnatal life, as documented in two instances in the literature [5,9], leading to the development of extra-renal RCC. 

In our case, we present an exceptionally rare occurrence of clear cell RCC with normal bilateral kidneys, where the tumor's origin can be traced back to a delayed neoplastic transformation in mesonephric embryonal remnants.

Similar types of neoplasms are extremely rare and are estimated in the literature to have developed primarily in mesodermal embryonic remnants. In the literature [5,9] as well as in our case the tumor was found incidentally. As it seems, the extra-renal RCC is asymptomatic and the diagnosis is based on imaging and histopathology. 

## Conclusions

We report a rare case of extra-renal clear cell RCC in the right colonic fossa. The origin of the tumor can be attributed to a delayed neoplastic transformation in mesonephric embryonal remnants, as the mass did not have any relationship with the kidneys. Clinicians should be aware of this rare entity as its diagnosis is challenging and is based on pathology.

## References

[1] Capitanio U, Bensalah K, Bex A (2019). Epidemiology of renal cell carcinoma. Eur Urol.

[2] Moch H, Cubilla AL, Humphrey PA, Reuter VE, Ulbright TM (2016). The 2016 WHO Classification of tumours of the urinary system and male genital organs-part A: renal, penile, and testicular tumours. Eur Urol.

[3] (2024). Renal cell carcinoma. EAU Guidelines.

[4] Hasan R, Kumar S, Monappa V, Ayachit A (2015). Primary extra-renal clear cell renal cell carcinoma masquerading as an adrenal mass: a diagnostic challenge. Urol Ann.

[5] Terada T (2012). Extra-renal clear cell renal cell carcinoma probably arising from mesodermal embryonic remnants. Pathol Int.

[6] Jayson M, Sanders H (1998). Increased incidence of serendipitously discovered renal cell carcinoma. Urology.

[7] Israel GM, Bosniak MA (2005). How I do it: evaluating renal masses. Radiology.

[8] Potter EL (1972). Normal and Abnormal Development of the Kidney. https://books.google.gr/books/about/Normal_and_Abnormal_Development_of_the_K.html?id=qhBsAAAAMAAJ&redir_esc=y.

[9] Nunes G, Pinto-Marques P, Sequeira P, Mendonça E (2019). Primary extrarenal renal cell carcinoma: a unique diagnosis performed through endoscopic ultrasound. GE Port J Gastroenterol.

